# Analysis of Primary Iris and Ciliary Body Cysts in Chinese Patients With Primary Angle Closure Disease

**DOI:** 10.3389/fmed.2021.704200

**Published:** 2021-08-26

**Authors:** Kun Wei, Chengguo Zuo, Jinyi Xu, Liming Chen, Dingqiao Wang, Zhongshu Tang, Mingkai Lin

**Affiliations:** State Key Laboratory of Ophthalmology, Zhongshan Ophthalmic Center, Sun Yat-sen University, Guangzhou, China

**Keywords:** PACD, primary iris and ciliary body cysts, UBM, monocular, glaucoma

## Abstract

**Aim:** To investigate the incidence and clinical features of primary iris and ciliary body cysts in Chinese primary angle closure disease (PACD). Patients were evaluated by measuring and analyzing the cysts with an ultrasound biomicroscope (UBM).

**Methods:** The data of patients diagnosed with PACD were reviewed. Demographic data were collected, and the cyst number, size, location, and trabecular-iris angle (TIA) were measured, with the size including the longest diameter (LD) and its corresponding vertical diameter (CVD).

**Results:** A total of 1,334 cases (2,317 eyes) were reviewed, and 409 cysts were found in 131 cases (168 eyes), with an average of 2.43 ± 3.14 cysts per eye. The ages of the patients with cysts ranged from 25 to 80 years, with an average age of 55.24 ± 12.22 years. The detection rate was 7.3%, and the majority of cysts were located in the iridociliary sulcus. Among the 131 patients, 94 had monocular cysts, while binocular cysts occurred in 37 patients. The locations of the cysts in both eyes were mainly in the inferior and temporal quadrants (42.5 and 34.0%, respectively). The cysts were mainly of medium size (49.9%), followed by small cysts (33.3%), large cysts (14.7%) and giant cysts (2.2%). The average LD was 0.68 ± 0.33 mm, and the average CVD was 0.45 ± 0.23 mm. There were no statistically significant differences in the TIA between the cyst area and unaffected area.

**Conclusions:** The incidence of cysts is 7.3% in the PACD population. The cysts are mainly monocular, medium in size, and located in the iridociliary sulcus. Additionally, the cysts were located mainly in the inferior and temporal quadrants. These cysts have little effect on the anterior chamber angle.

## Introduction

Glaucoma ranks among the top three blinding eye diseases in the world, and the number of cases is on the rise, according to recent surveys. Investigators predict that the number of glaucoma patients in China will increase to approximately 25 million by 2050 (1). Primary angle closure disease (PACD) is a major blinding disease in the Asian population, and PACD includes primary angle closure suspect (PACS), primary angle closure (PAC) and primary angle closure glaucoma (PACG). The structure and position of the iris and ciliary body are important anatomical structures in the pathogenesis of PACD (2, 3). The diagnosis and treatment effect may be affected by abnormalities of the iris and ciliary body. One of the most important congenital/primary abnormalities includes primary iris and ciliary body cysts, which are located mainly in the iridociliary sulcus or ciliary body (4–6). However, to the best of our knowledge, there are no relevant systematic studies on primary iris and ciliary body cysts in PACD patients.

The aim of this study was to investigate the incidence and clinical features of iris and ciliary body cysts in Chinese PACD patients by analyzing ultrasound biomicroscope (UBM) data.

## Materials and Methods

### Methods

Patients diagnosed with PACD underwent UBM anterior segment examination at the Zhongshan Ophthalmic Center of Sun Yat-sen University. We recruited patients with PACD who met the following criteria: age over 18 years, diagnosis of PACD, and intraocular pressure (IOP) controlled within the normal range for more than 1 week. The definitions of PACG, PACS and PAC followed those of the International Society of Geographical & Epidemiological Ophthalmology (ISGEO) (7). Patients with corneal disease, previous ocular surgery, ocular trauma, ocular inflammation, or severe systemic disease were excluded. This study was approved by the Ethics Review Committee (NO. 2021KYPJ003).

### Diagnostic Classification

**Figure 1:**
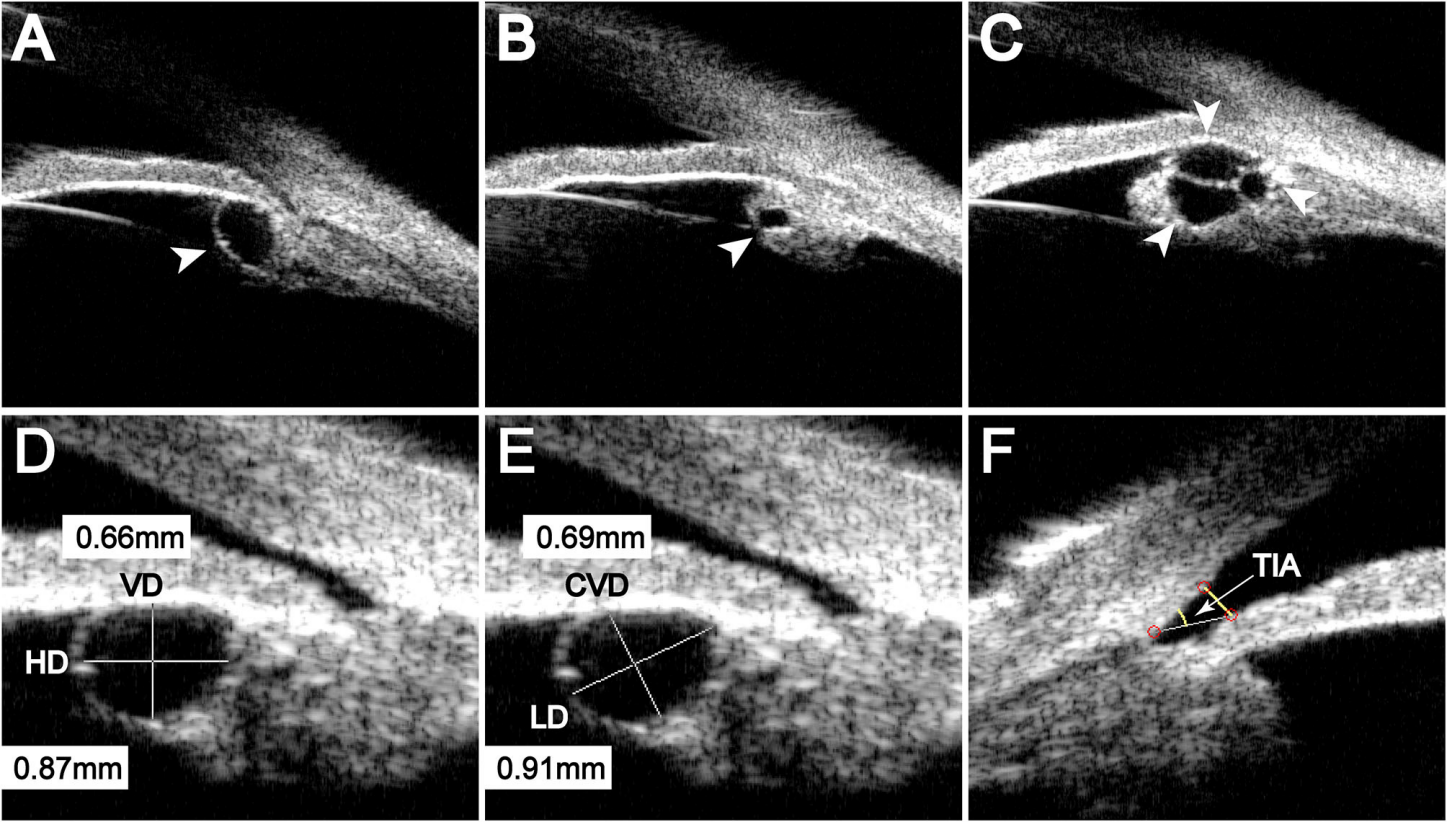
The type and measurement of the cysts. **(A)** Cyst located in the iridociliary sulcus, **(B)** Cyst located in the ciliary body, **(C)** Multiple cysts. **(D)** and **(E)** Different measurement methods for the same cyst; HD, horizontal diameter; VD, vertical diameter; LD, longest diameter; CVD, corresponding vertical diameter of the LD. **(F)** TIA, the trabecular-iris angle.

Single cyst refers to only one cyst being found in one eye (Figures 1A,B). Multiple cysts refers to two or more cysts being found in one eye (Figure 1C). Monocular multiple cysts (MMCs) refers to multiple cysts in only one eye with the other having no cysts. Bilateral multiple cysts (BMCs) refers to multiple cysts in both eyes. Monocular single cysts (MSCs) indicates that only one eye had a single cyst with the other having no cysts. Bilateral single cysts (BSC) indicates a single cyst in each eye. Bilateral mix type cysts (BMTC) refers to a single cyst in one eye and multiple cysts in the other eye. The cyst size was determined by calculating its mean size (MS: average of the longest diameter (LD) and its corresponding vertical diameter (CVD) with the UBM); a MS <0.4 mm was defined as a small cyst, a MS ranging from 0.4 to 0.8 mm was defined as a medium cyst, a MS ranging from 0.8 to 1.3 mm was defined as a large cyst, and a MS >1.3 mm was defined as a giant cyst (8).

### Examination Methods

The UBM (model SW-3200 L; Tianjin Suowei Electronic Technology Co, Ltd., Tianjin, China) in the Glaucoma Department of Zhongshan Ophthalmic Center of Sun Yat-sen University was used for inspection. During the examination, the examinee was asked to lie in the supine position. After the examinee was fully anesthetized with topical anesthesia (0.5% proparacaine hydrochloride), the eye cup without the fundus was placed in the conjunctival sac, and an appropriate amount of sterile normal saline was added. The examiner held the UBM probe on one hand and examined the eye from 12 o'clock clockwise around its entire circumference. Two-dimensional images of the anterior segment of the eyes were recorded, and a clear image was selected for preservation.

### Analysis Methods

UBM software was used to measure the cyst, and measurements included the affected eye and the position, LD and CVD of the cyst. The average value and the number of cysts in each eye were also calculated (6, 9). To avoid deviations caused by interobserver error, all measurements were conducted by a single experienced operator. LD represented the longest axis of the cyst, and CVD represented the longest axis perpendicular to the LD (Figure 1E). The trabecular-iris angle (TIA) was measured using the ACA/AOD function in the UBM software, with the apex at the scleral spur and the arms of the angle passing through a point on the trabecular meshwork 500 μm from the scleral spur and a perpendicular point on the iris (Figure 1F).

### Statistical Analysis

All data were analyzed using the statistical software SPSS 23. Differences in mean values were examined using *t*-tests. Categorical data were analyzed using the chi-square test. The incidence of cysts was calculated by means of the odds ratio. A *P*-value of <0.05 was considered to indicate statistical significance. There was no significant difference if the 95% CI of the odds ratio included 1.

## Result

### Detection Rate and General Situation of the Primary Iris and Ciliary Body Cysts

The data of 1,334 patients (2,317 eyes), including 499 males (818 eyes) and 835 females (1,499 eyes), were reviewed. Their ages ranged from 19 to 94 years, with an average age of 60.41 ± 12.07 years. There were 824 cases (1,193 eyes) with PACS, 367 cases (448 eyes) with PAC and 535 cases (676 eyes) with PACG. Of the 1,334 cases (2,317 eyes), 131 cases (168 eyes), aged 25–80 years (average age of 55.24 ± 12.22 years), were found to have primary iris and ciliary body cysts, and the average detection rate accounted for 7.3% of the eyes examined. Of all 131 PACD patients with cysts, 54 (72 eyes) were males, with a 10.8% detection rate in the male population, and 77 (96 eyes) were females, with a 9.2% detection rate in the female population. The odds ratio was 1.195 (95% CI: 0.828–1.724), which indicated that there was no significant difference in the incidences of males and females. Among them, 59 patients (68 eyes) had PACS, 43 patients (46 eyes) had PAC, and 44 patients (54 eyes) had PACG (Table 1). There was no significant difference in the proportion of cysts in different diseases (*P* = 0.180).

**Table 1:** Demographic features of the population with cysts.

	**Disease (eye)**			
**AGE**	**PAC**	**PACG**	**PACS**	**Total**	**Proportion**
≤ 29	2	0	1	3	1.8%
30–39	4	3	8	15	8.9%
40–49	12	14	12	38	22.6%
50–59	10	20	14	44	26.2%
60–69	13	8	23	44	26.2%
≥70	5	9	10	24	14.3%
Total	46	54	68	168	100%

### Distribution of the Cysts

Among the 2,317 eyes reviewed, we found 409 cysts in 168 eyes, with an average of 2.43 ± 3.14 per eye. Among the 409 cysts detected, 199 cysts were found in the right eye, and 210 cysts were found in the left eye. The proportion of cysts was not significantly correlated with the laterality of the eye (*P* = 0.475) (Table 2).

**Table 2:** The morphology of the cysts.

	**Males**				**Females**			
	**OD**		**OS**		**OD**		**OS**	
	***N***	**%**	***N***	**%**	***N***	**%**	***N***	**%**
Small	28	28.6%	34	35.4%	38	37.6%	36	31.6%
Medium	47	48.0%	45	46.9%	48	47.5%	64	56.1%
Large	19	19.4%	15	15.6%	12	11.9%	14	12.3%
Giant	4	4.1%	2	2.1%	3	3.0%	0	0.0%
Total	98	100.0%	96	100.0%	101	100.0%	114	100.0%

Of 409 cysts, 283 (69.2%) were located in the iridociliary sulcus (Figure 1A), and 126 (30.8%) were located in the ciliary body (Figure 1B). The location of cysts was not significantly related to sex (*P* = 0.632) or the laterality of the eye (*P* = 0.479).

**Figure 2:**
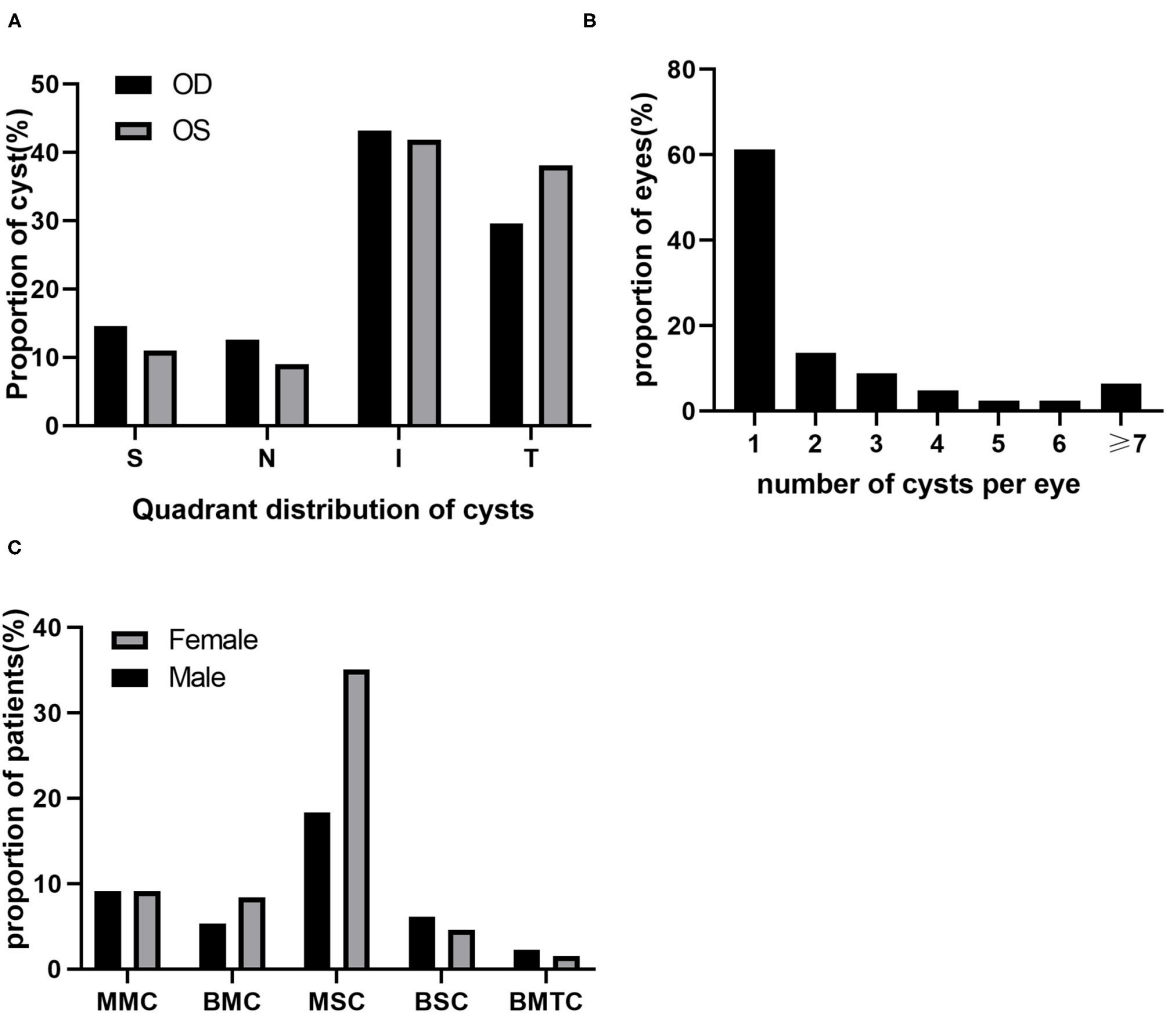
Distribution of cysts. **(A)** Quadrant distribution of cysts; N, nasal; I, inferior; T, temporal; S, superior. **(B)** Number of cysts per eye. **(C)** Type of cysts; MMC, monocular multiple cysts; BMC, bilateral multiple cysts; MSC, monocular single cysts; BSC, bilateral single cysts; BMTC, bilateral mix type cysts.

Among the 409 cysts, the most common quadrant was the inferior quadrant (42.5%, 174 cysts), followed by the temporal quadrant (34%, 139 cysts), superior quadrant (12.7%, 52 cysts) and nasal quadrant (10.8%, 44 cysts), indicating that most of the cysts occurred in the inferior and temporal quadrants (*P* < 0.001) (Figure 2A). We also found that there was no significant difference in the distribution of cysts in the left and right eyes (*P* = 0.221).

Among 168 eyes with cysts detected, 103 (61.3%) eyes had single cysts, and 65 (38.7%) eyes had multiple cysts. Multiple cysts with 2 to 3 per eye were common, accounting for 22.6% of eyes. Eyes with more than 4 cysts were less frequent, accounting for 16.1% of eyes (Figure 2B). Among the 131 patients, 94 had monocular cysts, and 37 had binocular cysts. The incidence of MSCs was significantly higher than that of other types (*P* < 0.001). Additionally, we found that the proportion of females with this type was higher than that of males (Figure 2C).

### Morphological Analysis of Cysts

Among 409 cysts, the average LD was 0.68 ± 0.33 mm, ranging from 0.18 to 2.07 mm, and the average CVD was 0.45 ± 0.23 mm, ranging from 0.08 to 2.04 mm. There was no statistically significant difference between the left and right eyes (*P*_LD_ = 0.192, *P*_CVD_ = 0.145).

Among the cysts detected, most were medium cysts, followed by small cysts and large cysts, and the least common were giant cysts. The size distribution of the cysts showed no significant difference between the laterality of eyes (*P* = 0.312) or between males and females (*P* = 0.263) (Table 2).

### Analysis of the Effect of Cysts on the Anterior Chamber Angle

To analyze the correlation between the cyst and angle more accurately, we measured the TIA of the cysts and the affected eyes. The mean values of TIA at the 3, 6, 9 and 12 o'clock positions were used as the baseline TIA of the affected eye. If there was a cyst at the abovementioned clock position, the average value of the other three clock positions was taken as the baseline TIA. After analyzing the relevant data by means of SPSS software, we found that there was no significant difference in TIA between the area of the cysts and the affected eyes (*P* = 0.236) (Table 3).

**Table 3:** Analysis of the correlation between cysts and the anterior chamber angle.

**Cyst location**	**Cyst TIA**	**Baseline TIA**	***P***
Ciliary body	13.25 ± 14.51	13.12 ± 12.85	0.896
Iridociliary	10.12 ± 11.81	11.03 ± 10.35	0.145
Total	10.97 ± 12.65	11.60 ± 11.10	0.236

## Discussion

Using UBM for inspection of 1,334 patients (2,317 eyes) who met our criteria, we found that there were a total of 131 cases (168 eyes) with primary iris and ciliary body cysts. Single cysts accounted for 61.3% of cases, medium cysts accounted for 49.9%, iridociliary sulcus cysts accounted for 69.2%, and inferior quadrant cysts and temporal quadrant cysts accounted for 42.5 and 34.0%, respectively. These results indicate that the incidence of primary iris and ciliary body cysts in Chinese PACD is 7.3% and that the cysts are mainly monocular, medium in size, distributed in the iridociliary sulcus and located in the inferior and temporal quadrants. Additionally, the presence of a cyst does not lead to significant narrowing of the anterior chamber angle.

Despite the critical role of the structure and position of the primary iris and ciliary body in the pathogenesis of PACD, few relevant systematic studies with large samples are currently available. This may be related to the low detection rate of cysts in routine ophthalmic examinations due to iris occlusion (10). In our study, with the application of UBM, ciliary body cysts were observed thoroughly. Since primary iris and ciliary body cysts are mostly congenital (4, 5), the prevalence should be independent of age and eye disease. Nevertheless, the cysts we detected were significantly related to age (89.3% were present in patients over 40 years old). This incidence may be related to the fact that patients with PACD are more likely to come to the hospital for examination due to eye discomfort and thus have a higher probability of cysts screened by UBM. Our data showed that patients with cysts were mainly 40–79 years old, which was also the peak onset age of PACD (11).

With regard to the incidence of cysts, the data obtained by different researchers varied greatly (6, 9, 11). After carefully analyzing the respective research methods and survey populations, we speculated that the differences in incidence may be due to race, age and disease characteristics of the subjects. Different types of UBMs and detection methods would also have an impact. This view is consistent with Kunimatsu, S's article (6).

Our data showed that the cysts were located mainly in the inferior quadrant, followed by the temporal quadrant, superior quadrant and nasal quadrant, which was consistent with the results of other researchers (6, 9, 11). In traditional filtration surgery, antiglaucoma surgery is less affected by the cyst, as the surgical areas are concentrated mostly in the upper area. In the clinic, lens extraction is widely used to treat primary angle closure glaucoma (12, 13). Cysts may have a significant influence on lens extraction, as doctors usually operate in the temporal area (14, 15). Therefore, the presence of cysts in the surgical area may increase the difficulty of an operation when the patient needs lens extraction. For some reasons, a proportion of patients have to undergo antiglaucoma surgery on the inferior and nasal sides due to uncontrolled IOP after the initial antiglaucoma surgery fails (16–18). The presence of cysts may make surgery more difficult. Attention should be given to the situation and type of ocular cysts, especially whether there are cysts in the operation area, which is conducive to reducing the incidence of intraoperative and postoperative complications. A researcher once reported a case in which cyst blockage caused iridotomy failure (19). Another researcher also believes that after precise positioning of the cyst location, selecting a cyst-free area for laser peripheral iridotomy can achieve better surgical results (20).

Some researchers have used horizontal and vertical diameters to measure cyst size (8). In our research, we found that most cysts were irregular in shape and changeable in position. For these cysts, traditional measurements could not accurately reflect the size of the cyst (Figure 1D), so we used LD and CVD to measure the cysts (Figure 1E). We believe that this new method can more accurately reflect the size of the cyst.

For PACD patients, the degree of narrowing or closure of the anterior chamber angle directly affects the diagnosis and treatment of glaucoma. To further explore the relationship between the cyst and angle, we compared the TIA between the clock position of the cyst and the affected eye, and it turned out that there was no significant correlation between the TIA of the cyst and the baseline TIA of the eye (*P* = 0.236). A few studies have found that cysts might push the iris root closer to the anterior chamber angle, resulting in narrower angles or closure (21, 22). Some researchers believe that cysts can lead to secondary angle closure or even acute secondary glaucoma (23, 24). However, these reports are about large or giant cysts. Since our study identified mainly small and medium cysts, no statistical significance was found in this relatively large cohort, and many studies have shown similar results (8, 25). When PACD coexists with cysts, the possibility of aggravated glaucoma is relatively low.

## Limitations

Bias may exist in hospital-based cross-sectional studies. A larger sample of follow-up observations is essential to verify these results before a more accurate conclusion can be drawn.

Our study shows that the incidence of primary iris and ciliary body cysts in Chinese patients with PACD is 7.3%. Most of them are MSCs. The cysts were mainly medium cysts, with an average size of 0.68 ± 0.33 mm in LD and 0.45 ± 0.23 mm in CVD. Moreover, most of the cysts are located in the inferior and temporal quadrants, which may easily affect an operation on the inferior temporal side. When a patient needs antiglaucoma surgery, the precise positioning of the iris and ciliary body cysts before surgery can help reduce intraoperative and postoperative complications. Additionally, the presence of a cyst does not lead to significant narrowing of the anterior chamber angle. For non-surgical PACD patients, the presence of cysts does not require special treatment.

## Data Availability Statement

The original contributions generated for the study are included in the article/supplementary material, further inquiries can be directed to the corresponding author/s.

## Ethics Statement

The studies involving human participants were reviewed and approved by Ethics Review Committee of Zhongshan Ophthalmic Center, Ethical number: 2021KYPJ003. The patients/participants provided their written informed consent to participate in this study.

## Author Contributions

ML and CZ: design and conduct of the study. LC: collection of the data. KW and ZT: analysis the data. KW, CZ, JX and DW: preparation of the manuscript. KW, CZ, JX, LC, DW, ZT, and ML: review and final approval of the manuscript. All authors contributed to the article and approved the submitted version.

## Conflict of Interest

The authors declare that the research was conducted in the absence of any commercial or financial relationships that could be construed as a potential conflict of interest.

## Publisher's Note

All claims expressed in this article are solely those of the authors and do not necessarily represent those of their affiliated organizations, or those of the publisher, the editors and the reviewers. Any product that may be evaluated in this article, or claim that may be made by its manufacturer, is not guaranteed or endorsed by the publisher.
